# Distinct selectivity inside self-assembled coordination cages

**DOI:** 10.3389/fchem.2023.1269471

**Published:** 2023-09-05

**Authors:** Yang Liang, Xiaojuan Zhou, Sreeraj Gopi, Rui Wang

**Affiliations:** Center for Supramolecular Chemistry and Catalysis and Department of Chemistry, College of Science, Shanghai University, Shanghai, China

**Keywords:** distinct selectivity, self-assembled coordination cages, constrained environment, confined spaces, noncovalent protecting group

## Abstract

Supramolecular containers have long been applied to regulate organic reactions with distinct selectivity, owing to their diverse functions such as the ability to pose a guest molecule(s) with a certain orientation and conformation. In this review, we try to illustrate how self-assembled coordination cages could achieve this goal. Two representative cage hosts, namely, self-assembled Pd(II)-ligand octahedral coordination cages ([Pd_6_L_4_]^12+^) and self-assembled Ga(III)-ligand tetrahedral coordination cages ([Ga_4_L_6_]^12−^) are selected as the pilot hosts that this mini review covers. Representative works in this area are presented here in brief.

## 1 Introduction

Ever since its establishment, supramolecular chemistry has received enormous attention and has rapidly become one of the most important fields in modern chemistry (Lehn, 1993; Lehn, 1995; Housecroft, 2021). Depending on various non-covalent interactions, supramolecular chemistry has been thoroughly studied and applied to various research areas, including molecular recognition, molecular devices, nanochemistry, and catalysis, etc. (Vriezema et al., 2005; Ariga and Kunitake, 2006; van Leeuwen, 2008; Kolesnichenko and Anslyn, 2017). Within the field of supramolecular chemistry, molecular container compounds are large hollow molecules with inner cavities that can accommodate various guest molecules. The inner cavity (inner phase) of a molecular container provides an elegantly isolated hydrophobic microenvironment, resembling active enzyme receptor sites, which can pose a guest molecule(s) with a certain fixed orientation and conformation. Logically, chemists have tried to simulate the function of natural enzymes by developing various synthetic supramolecular containers in recent decades to tackle problems in traditional organic chemistry, including improving reactivity and reaction rate, inducing new reaction selectivity, and even producing new reaction pathways (Purse and Rebek, 2005; Koblenz and WassenaarReek, 2008; Hooley and Rebek, 2009; Murase and Fujita, 2010; Yu and Rebek, 2018; Morimoto et al., 2020; Olivo et al., 2021). Selectivity is a crucial factor in conducting a certain organic reaction, but still remains one of the most significant challenges in organic synthetic chemistry (Ward, 1999). Poor reaction selectivity always results in complicated and even unachievable separation and purification procedures, which degrade the economy and efficiency of the synthetic methodology. It is rather difficult to regulate and control the reaction selectivity because the difference between transition state free energies regarding electronic, steric, and stereoelectronic influence of related reaction pathways, which give rise to isomeric products, is small (Chao et al., 1991; Balcells et al., 2016). Organic chemists have long made great efforts trying to regulate reaction selectivity (Neufeldt and Sanford, 2012; Davis and Phipps, 2017), and supramolecular chemistry and containers have come into their sights. Completely different from a bulk solution, the inner microenvironment of a supramolecular container can isolate and protect the guest substrates from the outside media, with certain fixed orientation and configuration achieved through various noncovalent interactions This certainly affects and controls the emergence of different or new selectivity as well as products that are not the major outcome or that even cannot be detected in regular conditions by altering the corresponding transition state free energies and reaction processes (Purse and Rebek, 2005; Koblenz and WassenaarReek, 2008; Hooley and Rebek, 2009; Murase and Fujita, 2010; Yu and Rebek, 2018; Morimoto et al., 2020; Olivo et al., 2021). Self-assembled coordination cage is a very important category of supramolecular containers, and, different from covalently constructed macrocycle hosts, they are readily self-assembled from ligands and metal ions through noncovalent coordination interactions (Murase and Fujita, 2010; García-Simón et al., 2014; García-Simón et al., 2015; Cullen et al., 2016; Howlader et al., 2016; Guo et al., 2017; Ueda et al., 2017; Hong et al., 2018; Martí-Centelles et al., 2018; Yu et al., 2018). The ionic property of self-assembled coordination cages provides them with two main advantages: (1) water solubility, which facilitates them in achieving molecular recognition and catalysis in water and (2) the ability to preferably accommodate and catalyze ionic guests and reactions. The Fujita group (Murase and Fujita, 2010) and the Toste, Raymond, and Bergman groups (García-Simón et al., 2014) have done a lot of pioneering and seminal works in this research area and have produced various fruitful achievements. In this mini review, we focus on summarizing representative examples of organic reactions inside self-assembled coordination cages that show distinct selectivity from that of the outside bulk solution conditions. Two representative types of self-assembled coordination cages were selected, namely, self-assembled Pd(II)-ligand octahedral coordination cages and self-assembled Ga(III)-ligand tetrahedral coordination cages, and this mini review is classified according to them.

## 2 Mini review

### 2.1 Self-assembled Pd(II)-ligand octahedral coordination cage ([Pd_6_L_4_]^12+^)

**FIGURE 1:**
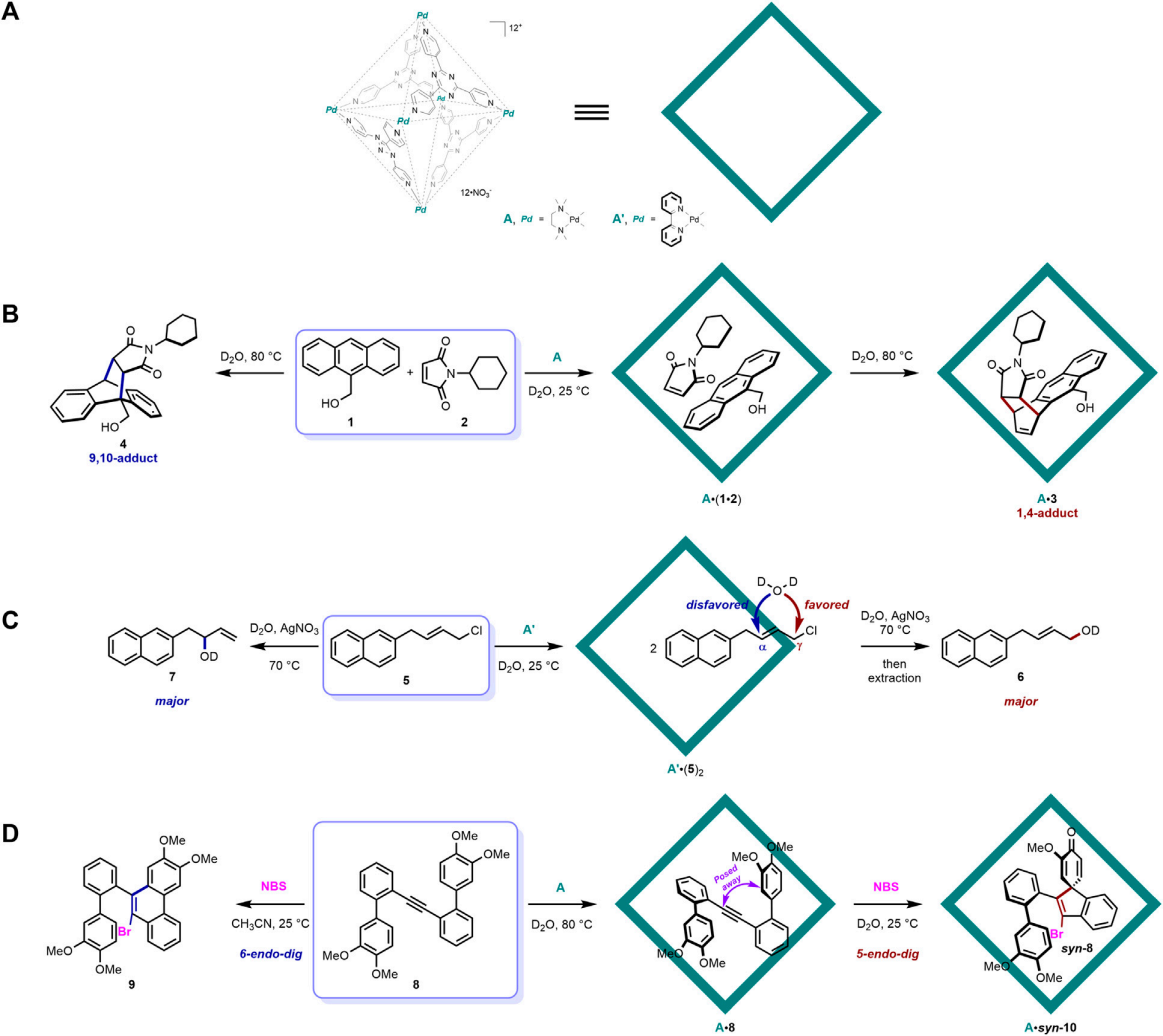
Examples of [Pd_6_L_4_]^12+^ cage mediated organic reactions with distinct selectivity: **(A)** structure of the representative self-assembled Pd(II)-ligand octahedral coordination cage; **(B)** cage host-mediated Diels-Alder reaction with distinct selectivity; **(C)** cage host-mediated nucleophilic substitution reaction of allylic chlorides with preferable terminal site selectivity; **(D)** cage host-mediated electrophilic spirocyclization of a 2-biphenylacetylene with distinct selectivity.

The self-assembled Pd(II)-ligand octahedral coordination cage was initially developed by the Fujita group (Fujita et al., 1995) and has been applied to various molecular recognition study and reaction catalysis research since then (Murase and Fujita, 2010). As shown in Figure 1A, it is a hollow octahedral framework self-assembled from six Pd(II) ions and four organic ligands, with a triazine core and three substituted 4-pyridyl groups. This cage host possesses 12 positive charges, which renders it very water soluble. The electron-deficient nature of the aromatic panel ligands endows it with a better ability to recognize electron-rich guest molecules and a significant role as a photosensitizer to facilitate guest-to-host photoinduced electron transfer (PET) (Yoshizawa et al., 2004; Furutani et al., 2009; Murase et al., 2011; Murase et al., 2012; Cullen et al., 2019). The Fujita group has reported plenty of organic reaction regulation works utilizing this distinguished supramolecular cage host (Murase and Fujita, 2010).

In 2006, Fujita and co-workers reported a seminal work on the Diels–Alder reaction between anthracene and phthalimide inside the [Pd_6_L_4_]^12+^ cage host with distinct site selectivity (Yoshizawa et al., 2006) (Figure 1B). Owing to its high localization of π-electron density at the center ring (Cheng and Li, 2003), the Diels–Alder cyclization reaction of anthracene generally occurs at this site and gives rise to a 9,10-adduct that bridges the center ring of the anthracene framework (Breslow, 1991; Fringuelli and Taticchi, 2002; Stuhlmann and Jäschke, 2002). However, when the corresponding reaction of 9-hydroxymethylanthrancen (**1**) and *N*-cyclohexylphthalimide (**2**) was moved into the cavity of the [Pd_6_L_4_]^12+^ cage host **A** in Fujita’s report, the site selectivity of this Diels–Alder reaction was altered to the 1,4-position of anthracene, producing a *syn*-isomer of 1,4-adduct **3**. X-ray crystallographic analysis further confirmed the structure of the product together with its orientation and conformation inside the cage host, revealing that the naphthalene ring of the product interacts closely with one of the triazine ligand of **A** through π-π stacking. In the following control experiment without **A**, only the conventional 9,10-adduct **4** was detected without any **3**. The fixed orientation of the substrates inside the cage host was responsible for this distinct site selectivity of the 1,4-position. In the force-field calculation study, **2** was shown to be parallel to **1**, with its double bond in close contact with the 1,4- but not the 9,10-position of **1**, owing to the steric effect inside the cage host. In another comparable example, when a less sterically demanding *N*-propylphthalimide substrate was applied, only the 9,10-adduct was formed, indicating the crucial role of the steric bulkiness of the *N*-substituent on the dienophile inside the cage host. This pioneering work perfectly validated the ability of supramolecular containers to accommodate guest substrates inside and to lend them certain fixed orientations and conformations, which give rise to a distinct reaction selectivity that is not seen in traditional bulk solution conditions. In following research, the Fujita group further provided a series of novel pericyclic reactions inside a similar cage host with distinct and controllable selectivity (Murase and Fujita, 2010).

In 2012, another nucleophilic substitution reaction of allylic chlorides inside a cage host with preferable terminal site selectivity was reported by Fujita and co-workers (Kohyama et al., 2012) (Figure 1C). Generally, the reaction of allylic chlorides with nucleophiles takes place at both the α- and γ-positions, and the steric and electronic effects of substrates together with the polarity of the solvent collectively influence the ratio of α- and γ-products (DeWolfe and Young, 1956). The cage host **A′** also proved to be effective in recognizing the allylic chloride substrate (**5**) inside its hydrophobic cavity. In this mode, the α-position was buried inside the cavity, with the γ-position pointed out of the inner phase of **A’**. In this reaction, the solvent D_2_O acted as the nucleophile, and the hydrophobic pocket of **A′** prevented its entering and protected the buried α-reactive position from contacting the incoming water nucleophile. On the other hand, the pointed-out terminal γ-position was exposed to the outside aqueous solution and was attacked by D_2_O, producing a terminal-induced product (**6**). In the control experiment without **A’**, the internal α-product (**7**) was major. Even though the ratio between terminal/internal products was not significant in this work, it represents an early example of the noncovalent protecting group function of supramolecular containers. Protecting groups are very famous in organic chemistry for their powerful function of preventing certain selected functional groups from reacting with other reagents (Schelhaas and Waldmann, 1996; Isidro-Llobet et al., 2009; Klán et al., 2013). Generally, they are covalently attached to the target moieties through pre-functionalization prior to the formal reaction and are deprotected after. However, just like the above example, supramolecular containers have offered a very promising alternative way of protecting and shielding certain reactive groups through the *in situ* recognition of the target moieties in the inner pocket via noncovalent interactions. The protecting manner of supramolecular containers has two main advantages: (1) the noncovalent mode of this concept requires no additional pre-functionalization procedures and it is weak enough to allow the substrate to easily dissociate from the protective host template, without further complicated deprotection procedures and (2) incompatible functional groups in traditional covalent protecting procedures can be tolerated in the noncovalent protecting system provided by supramolecular containers because no covalent bond formation is required. The [Pd_6_L_4_]^12+^ cage host was further applied to another reaction acting as this kind of noncovalent protecting groups to achieve site selective control (Takezawa et al., 2019).

Very recently, the same group have provided the electrophilic spirocyclization of a 2-biphenylacetylene in the presence of an electrophile through the fixing of conformation confined to the cavity of the cage host (Takezawa et al., 2022) (Figure 1D). When treated with electrophiles, 2-biphenylacetylene (**8**) underwent two different cyclization pathways: (1) one at the *ortho* position via *6-endo-dig* cyclization to produce phenanthrene derivatives (**9**) (Goldfinger et al., 1997; Yamaguchi and Swager, 2001; Li et al., 2007; Dou et al., 2013) and (2) one at the *ipso* position via *5-endo-dig* cyclization to produce benzospiro (Vriezema et al., 2005; Ariga and Kunitake, 2006)decane derivatives (**10**) (Appel et al., 2003; Zhang and Larock, 2005; Yu et al., 2008; Dohi et al., 2011a; Dohi et al., 2011b; Tang et al., 2012). In normal solution conditions, the former pathway and the corresponding phenanthrene products are often observed, and, only when the electron density at the *ipso* position is much higher than that at the *ortho* position, will the latter be major (Appel et al., 2003; Zhang and Larock, 2005; Yu et al., 2008; Dohi et al., 2011a; Dohi et al., 2011b; Tang et al., 2012). To achieve distinct selectivity of the latter, the authors naturally introduced the [Pd_6_L_4_]^12+^ cage host **A** into this reaction system. **8** was readily accommodated into the hollow pocket of **A**, and, according to ^1^H NMR, UV/Vis, and X-ray crystallographic analysis, the confined guest molecule was tightly packed with a folded conformation inside the host. The electron-rich aromatic rings of **8** interacted with the electron-deficient panel ligand of the host through π-π stacking. On the other hand, the *ortho* reactive positions were posed significantly away from the acetylene carbons, indicating the abovementioned *6-endo-dig* cyclization may be suppressed. Indeed, treating the host-guest complex with electrophile NBS produced the spiro product **10** quantitatively, with single *syn*-diastereoselectivity. A control experiment revealed that **9** was formed through *6-endo-dig* cyclization without the cage host. This recent example again showed how the [Pd_6_L_4_]^12+^ cage host could perfectly pose the guest molecule with a certain fixed orientation and conformation to induce distinct selectivity.

### 2.2 Self-assembled Ga(III)-ligand tetrahedral coordination cage ([Ga_4_L_6_]^12−^)

**FIGURE 2:**
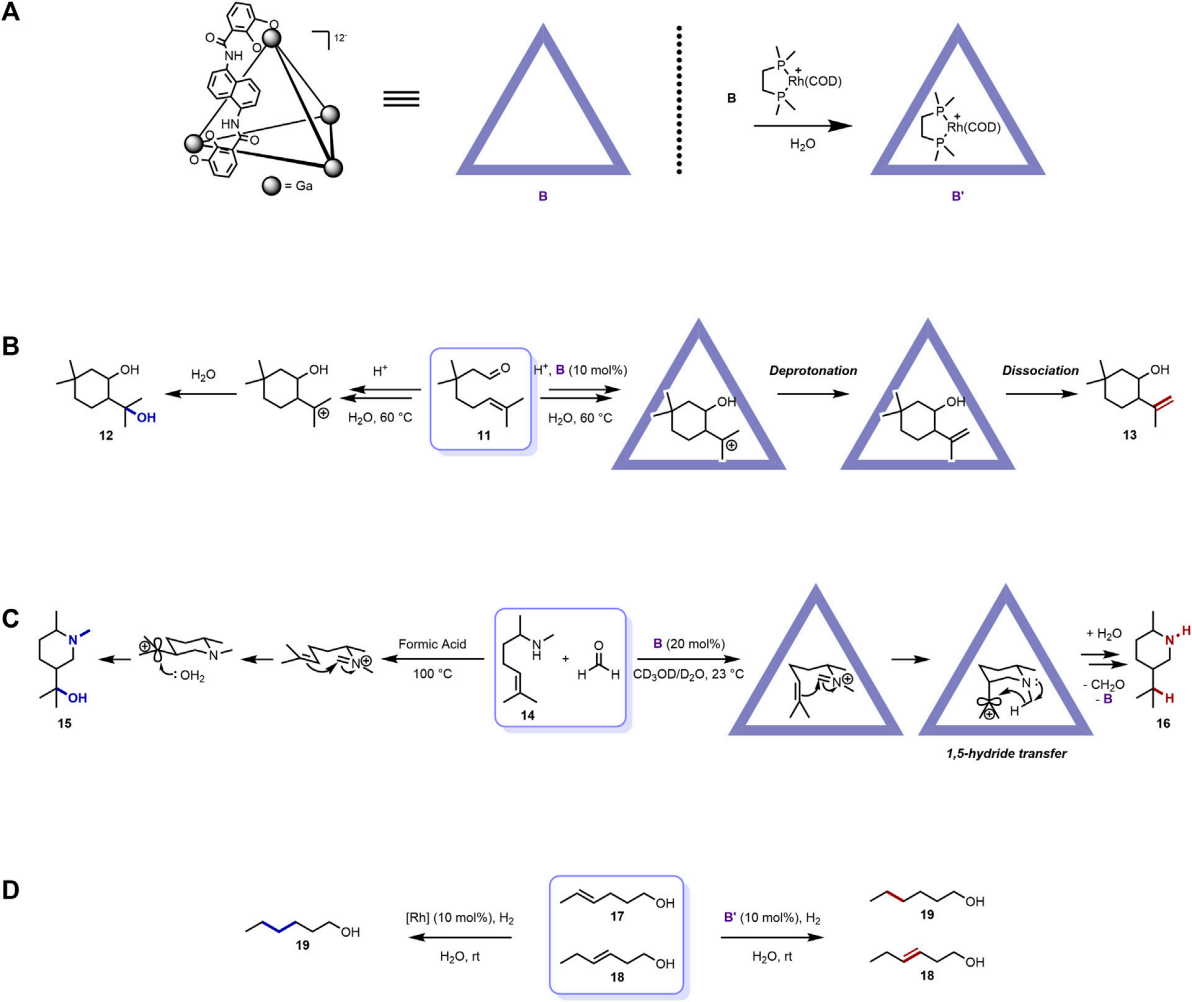
Examples of [Ga_4_L_6_]^12-^ cage mediated organic reactions with distinct selectivity: **(A)** representative structures of [Ga_4_L_6_]^12−^ cages; **(B)** cage mediated intramolecular Prins cyclization with distinct selectivity; **(C)** [Ga_4_L_6_]^12−^ cage mediated aza-Prins cyclization with distinct selectivity; **(D)** [Ga_4_L_6_]^12−^ cage mediated hydrogenation of olefins with distinct selectivity at the more terminal positions.

The self-assembled metal-ligand tetrahedral coordination cage (Figure 2A) was first reported by Raymond and co-workers (Caulder et al., 1998). It exhibits a tetrahedral shape and is spontaneously assembled from four metal ions, for example, Ga(III), Al(III), In(III), Fe(III), Ti(IV), Ge(IV), and six bis-bidentate catechol amide–containing ligands. Among them, the [Ga_4_L_6_]^12−^ cage has been widely used for mediating organic reactions. Similar to the [Pd_6_L_4_]^12+^ cage host, the ionic property also generates good water solubility for the [Ga_4_L_6_]^12−^ cage. On the other hand, the [Ga_4_L_6_]^12−^ cage host is excellent at recognizing cationic guest molecules because of its negative charge nature. The electron-rich character also renders the [Ga_4_L_6_]^12−^ cage host photosensitizer functionality and the ability to further induce host-to-guest photoinduced electron transfer (PET) (Dalton et al., 2015). Various seminal organic reaction regulation works involving this [Ga_4_L_6_]^12−^ cage host have been provided by the collaboration between the Toste, Raymond, and Bergman groups (Hong et al., 2018).

In 2012, Toste, Raymond, Bergman, and co-workers reported the catalytic intramolecular Prins cyclization of citronellal and its derivatives with distinct selectivity, which resembled the active sites of many terpene synthases (Hart-Cooper et al., 2012) (Figure 2B). When treated with buffered acidic solution, the citronellal derivative substrate (**11**) underwent cyclization to produce two main types of products, with the diol product (**12**) being major (Clark et al., 1984; Yuasa et al., 2000; Cheng et al., 2009). The authors then applied the [Ga_4_L_6_]^12−^ cage host **B** to this reactive system to investigate the possibility of selectivity regulation. Not surprisingly, the product distribution was shifted, with the isopulegol-like product (**13**) being the major through deprotonation instead of nucleophilic capture by water of the cationic intermediate. As illustrated above, the negative charge–containing cage host [Ga_4_L_6_]^12−^ can preferably recognize and stabilize cationic species on the one hand, while on the other, the inner space of **B** is hydrophobic and can prevent water from entering its interior to contact the encapsuled **11**. The former factor stabilized and extended the lifetime of the cationic Prins cyclization intermediate; the latter hindered the nucleophilic attack of water that gave rise to the formation of the hydroxyl group. This work demonstrated the selectivity regulation ability of the [Ga_4_L_6_]^12−^ cage host.

Later, the authors also reported another catalytic bimolecular aza-Prins cyclization inside the same cage host with new reactivity and selectivity (Kaphan et al., 2015) (Figure 2C). The reaction between the unsaturated amine substrate (**14**) and formaldehyde in formic acid bulk solution under reflux produced the alcohol product (**15**). In the reaction process, the amine group first condensed with formaldehyde to yield the iminium ion intermediate, which underwent aza-Prins cyclization and hydration. When **B** was incorporated into this system, however, a totally new pathway and product (**16**) arose under mild condition. The condensed iminium ion intermediate was subsequently encapsuled into the interior of the cage host with a constrictive spherical transition state, where the double bond was placed to an axial position owing to the steric inner microenvironment of the cavity. The iminium ion intermediate then cyclized to form the carbocation intermediate, followed by an unexpected transannular 1,5-hydride transfer process, which gave rise to **16** after hydrolysis. The subsequent systematic mechanism investigation revealed that the rate-limiting step of this special reaction was the encapsulation of the iminium ion intermediate and the unusual 1,5-hydride transfer process was supported through kinetic analysis and isotopic labeling studies. This unconventional reactivity and selectivity were due to the hydrophobic interior of the cage host, which prevents water from entering its inner space to attack the resulting carbocation, as well as the steric effect inside the cavity that poses the carbocation group to the axial position, close to the hydrogen of the carbon adjacent to the nitrogen, which favors the 1,5-hydride transfer process. This extraordinary example, in the authors’ words, “represents a rare example of such an extreme divergence of product selectivity observed within a catalytic metal-ligand supramolecular enzyme mimic” and “represents the most pronounced deviation in reactivity within a supramolecular catalyst to date.”

Recently, in 2019, the same collaboration groups reported the catalytic hydrogenation of olefins with distinct selectivity at the more terminal positions with the help of the [Ga_4_L_6_]^12−^ cage host (Bender et al., 2019) (Figure 2D). In this work, host **B** was first mixed with a rhodium complex to produce the rhodium-encapsulated supramolecular catalyst **B’**. To illustrate the distinct selectivity of this catalyst, a mixture of methyl- and ethyl-substituted alkene substrates (**17** and **18**, respectively) were added to the catalyst aqueous solution under a H_2_ atmosphere at room temperature. After 20 h, only **17** was reduced, with nearly full retention of **18**. However, with just the rhodium catalyst free in the bulk solution, both **17** and **18** were reduced to **19** within just 1 h. In this function mode, the reactive center of the catalyst was accommodated and buried inside the cavity, and only sterically accessible reactive sites (methyl substituted one) could enter the interior of the host to contact the rhodium catalyst center. In other words, the supramolecular container acted as a reaction flask for the catalyst and the reacting moiety, while the outside parts were “protected” from the reagent, which is in complete contrast to the abovementioned cases, where the supramolecular host functions as the noncovalent protecting groups. This beautiful example showed the diverse function of the promising supramolecular container for regulating distinct selectivity in various organic reactions.

### 2.3 Conclusion

To summarize, we have reviewed representative examples of how supramolecular containers, especially self-assembled coordination cages, could induce distinct selectivity in various organic reactions, different from that in the bulk solutions. The cage host is spontaneously assembled by metal ions and suitable ligands, with no need to form covalent bonds. It is water soluble, but its interior is highly hydrophobic and does not allow water into its cavity, which can generally alter the mechanism of water-associated processes and gives rise to distinct selectivity in these cases. On the other hand, the cage host can pose the encapsulated guest molecule(s) with certain fixed orientations and conformations to induce different site selectivity and even new reactivity. In some cases, the cage host acts as a noncovalent protecting group that shields certain parts of the substrate from the outside reagents, giving rise to distinct site selective products. In addition, the cage host could also function as a reaction vessel, and only the sterically accessible sites of the substrate could come inside the host to react with the catalyst center while the outside moieties could be “protected”. All the examples illustrated above demonstrate the powerful ability of self-assembled coordination cages in regulating reaction selectivity.
